# Impact of the COVID‐19 pandemic on inflammatory bowel disease: The role of emotional stress and social isolation

**DOI:** 10.1002/smi.3080

**Published:** 2021-07-26

**Authors:** Boukje Yentl Sundari Nass, Pauline Dibbets, C. Rob Markus

**Affiliations:** ^1^ Department of Neuropsychology and Psychopharmacology Faculty of Psychology and Neuroscience Maastricht University Maastricht The Netherlands; ^2^ Dr. Rath Health Foundation Heerlen The Netherlands; ^3^ Clinical Psychological Science Maastricht University Maastricht The Netherlands

**Keywords:** COVID‐19, emotional stress, inflammatory bowel disease

## Abstract

Inflammatory bowel disease (IBD) is a chronic health condition exacerbated by negative emotional stress experiences. In the current study, we examined whether the outbreak of the COVID‐19 pandemic coincided with an increase in stress experiences and accordingly an aggravation of disease activity in IBD patients. Sixty‐three IBD patients (30 Crohn's disease or CD, 33 ulcerative colitis) completed an online survey during the COVID‐19‐related lockdown, assessing clinical disease activity, disease‐related quality of life, presence of functional gastrointestinal symptoms, social isolation and stress experiences. Scores were then compared to pre‐lockdown baseline screening. The pandemic yielded a significant baseline‐to‐lockdown increase in emotional stress and social isolation. Stress increments, particularly those occasioned by interpersonal tension and excessive interpersonal proximity, were associated with a worsening of functional gastrointestinal symptoms. Exacerbations of loneliness coincided with an escalation of CD activity, functional gastrointestinal symptoms and a decline in subjective health. Lastly, COVID‐19 anxiety was significantly related to CD symptom severity and social dysfunction. The findings show that shifts in IBD expression are closely linked to changes in emotional stress experiences and interpersonal relatedness. As such, they contribute to a better understanding of inter‐individual differences in IBD progression and provide leads for therapeutic interventions.

## 1 INTRODUCTION

Crohn's disease (CD) and ulcerative colitis (UC), collectively known as inflammatory bowel disease (IBD), are chronic intestinal conditions with unknown aetiology and an unpredictable clinical course—hallmarked by periods of relatively quiescence alternated with acute flares (Hanauer et al., 2010). Aggressive, repetitive disease flares may bring about irreversible bowel damage, and—conditional upon their severity and duration—escalate the risk of extraintestinal manifestations, comorbid conditions (Argollo et al., 2019; Lewis et al., 2008; Park et al., 2019) and carcinogenesis (Herszenyi et al., 2007). As no cure is available, therapeutic strategies orient towards the control of flare‐ups and maintenance of symptom‐free intervals (Hanauer et al., 2010). While these medical treatments (e.g., TNF‐alpha inhibitors) have a good chance of inducing remissions, the course of IBD often remains progressive and disabling in nature. In fact, penetrating complications (i.e., stricture, fistula, abscess, perforation) are still ‘by far the most frequent ultimate expression of CD’ (Cosnes et al., 2002), requiring surgical intervention in 75% of CD and almost 50% of UC patients—half of whom show a post‐operative relapse within a few years (Cosnes et al., 2002; Hanauer et al., 2010; Peyrin‐Biroulet et al., 2010). Despite this disturbing prospect, there is a remarkable variability in disease progression and pace in which complications occur (Hanauer et al., 2010). To elucidate these inter‐individual differences in IBD evolution and better control continuously relapsing–remitting disease episodes, it is of high importance to map potential flare‐inducing factors.

One factor hypothesized to play a role in IBD disease activity is the experience of psychological stress, (Araki et al., 2020; Cámara et al., 2009; Hisamatsu et al., 2007; Maunder & Levenstein, 2008; Mawdsley & Rampton, 2005; Targownik et al., 2015), which is thought to aggravate IBD symptomatology by altering gastrointestinal (GI) motor, secretory and sensory function, promoting intestinal permeability and reinforcing inflammatory responses in the gut (Mawdsley & Rampton, 2005, 2006; Sajadinejad et al., 2012). Though the mechanisms underlying the link between negative stressful experiences and symptomatic IBD are not yet fully understood, it is increasingly recognized that both psychological and biological pathways play a role in stress‐induced alterations in IBD expression.

Physiologically, the immediate consequences of stress on intestinal health are believed to be mediated by neuroendocrine‐immune pathways belonging to the brain–gut axis (BGA), most notably the hypothalamus–pituitary–adrenal (HPA) axis and the sympathetic nervous system—both of which are potent immunoregulators (Bonaz & Bernstein, 2013; Mawdsley & Rampton, 2005). Additionally, IBD and persistent stress are increasingly associated with structural and functional changes in various limbic structures that— given their regulatory control over brain–gut (e.g., autonomic and endocrine) effector pathways—may culminate in an exacerbation of symptoms (Agostini, Benuzzi, et al., 2013; Agostini, Filippini, et al., 2013; Agostini et al., 2011, 2017; Bao et al., 2015; Hong et al., 2014; Rubio et al., 2016; Thomann et al., 2016, 2017; Vogt, 2013).

Psychologically, stress and overwhelming emotional experiences may affect intestinal health by inducing spontaneous changes in cognitive–perceptual processes, leading to attentional narrowing (Cisler et al., 2009; van Steenbergen et al., 2011) whereby perceptual processing of negative affective stimuli may take precedence over more adaptive environmental cues. Such abnormal involvement in threat processing may deepen the subjective experience of stress and perpetuate physiological (neuroendocrine and autonomic) arousal (O'Donovan et al., 2013), rendering individuals more vulnerable to gut discomfort and poorer health outcomes.

Taken together, the growing insight in psychoneuroimmuno modulation through the brain–gut axis—and the effect ofstress‐induced alterations in consciousness and limbic function thereon—provides us with a solid framework to better understand how mental states and social conditions drive GI disease activity. In this prospective study, we investigated whether the sudden outbreak of the new coronavirus disease in early 2019 (COVID‐19)—presumably due to its disruptive effects on daily life and/or routines—coincided with a shift (increase) in negative emotional stress experiences in CD and UC patients and, correspondingly, a change (increase) in IBD symptomatology. The COVID‐19 pandemic and social restrictions enacted by the government to limit the spread of the virus altered daily routines drastically, forced people to withdraw from social life and often subjected them to a sharp increase in feelings of distress. The flood of stressors thus occasioned by the pandemic was multifaceted in nature—ranging from COVID‐19‐related fears (fear of contamination, disease and death), educational and occupational stress, financial difficulties, interpersonal conflicts, and social isolation up to COVID‐19‐related traumatic experiences (e.g., dying loved ones) (Kowal et al., 2020; Kujawa et al., 2020; Salari et al., 2020; Shanahan et al., 2020; Taylor et al., 2020). As to IBD patients, it seems plausible that the outbreak of COVID‐19 has hit them particularly hard. On the one hand, they often hold the belief that IBD and IBD drugs (immunosuppressants) render them more vulnerable to COVID‐19 (D’Amico et al., 2020), which may predispose them to greater COVID‐19‐related anxiety. On the other hand—inasmuch the availability of social support proved instrumental in reducing IBD relapse risk (Garcia‐Sanjuan et al., 2016)—they may exhibit greater emotional and symptomatic reactivity to acutely imposed social constraints (which deny them social support and limit their access to health professionals).

In conclusion, IBD is a serious health problem known to be exacerbated by stress experiences. Many have experienced the COVID‐19 pandemic and subsequent actions taken by the government to flatten the virus contamination curve as highly aversive emotional experiences and important promotors of stress. In the current study, we investigate to what extent the pandemic was indeed associated with a shift in experiences of stress and interpersonal connectedness in IBD patients and a corresponding change in GI symptom severity. Here, we hypothesize that a lockdown‐related increase in experiences of stress and loneliness is accompanied by a worsening of symptomatic disease and a decrease in quality of life (QoL), whereas a decline thereof coincides with a remission of clinical disease and an improvement of QoL.

## 2 MATERIALS AND METHODS

### 2.1 Study sample

Between January and March 15 2020, a total of 135 patients with GI‐related diseases participated in a pending study on the relationship between adverse life experiences and GI complaints. All had been recruited through a gastroenterological university clinic (MUMC+) and patient organization in the Netherlands (Crohn and colitis NL; through advertisements on social media channels) and were recontacted towards the end of the first lockdown (end of May, first week of June) to participate in the current study. Out of all invitees, 85 persons responded, of which 74 completed the survey. Patients still awaiting an official diagnosis, suffering from a GI condition other than IBD, and/or infected with COVID‐19 were excluded, bringing the total to 63 participants (aged 19–62, *M = *39, SD* = *12.31), whereof 44 women (23 CD, 21 UC) and 19 men (7 CD, 12 UC). Of these, two patients (1 CD, 1 UC) suffered from depression and one (UC) from susceptibility to psychosis, with none of them using psychotropics drugs. In the remaining 60 patients, the use of psychotropic substances was reported on one occasion (by a CD patient: benzodiazepines and selective serotonin reuptake inhibitor).

### 2.2 Procedure

**FIGURE 1:**
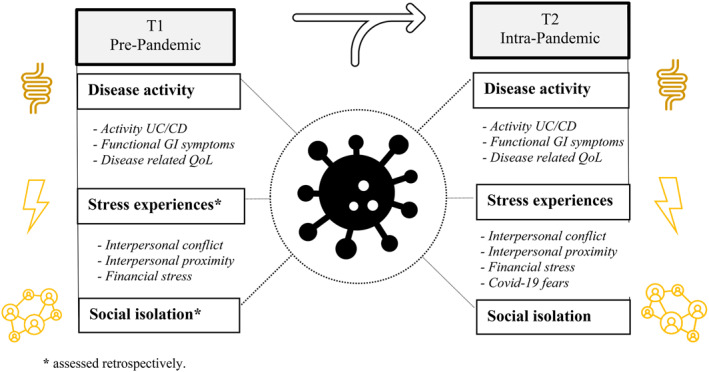
Schematic overview of the study design

Towards the end of the official lockdown, participants were invited by e‐mail to take part in the current study (see Figure 1). All had undergone basic pre‐lockdown screening (between January and March 15 2020), including baseline assessments of UC and CD disease activity, presence of functional GI symptoms and disease‐related QoL. Participants interested in taking part in the study received a link to the online survey (Qualtrics ®) and a personal access code. After having read the general outline of the study, participants marked the checkbox to give their informed consent. Next, the following questionnaires were presented in consecutive order: the self‐constructed corona and lockdown stress questionnaire, followed by the University of California Los Angeles (UCLA) loneliness scale, an index of irritable bowel syndrome symptom severity, the Inflammatory Bowel Disease Questionnaire, and two IBD clinical scoring indices. Upon completion, participants were debriefed. The study was approved by the Ethics Review Committee Psychology and Neuroscience at Maastricht University (ERCPN‐205_14_03_2019) in compliance with the Declaration of Helsinki. All participants gave their informed consent and none of them received financial compensation.

### 2.3 Measures

#### 2.3.1 Corona fear and lockdown‐related stress

As no validated COVID‐19 stress and anxiety questionnaire was readily available, a self‐constructed 12‐item questionnaire was used to measure four dimensions of self‐reported COVID‐19 and lockdown‐related stress. Factor 1—*Corona fears* in the past 2 months, was composed of four items, namely worries about getting infected, dying from corona, a loved one getting infected and increased risk of infection due to the presence of IBD. Reliability estimates for the subscale were high with a Cronbach's alpha of 0.86. Factors 2–4 assessed domestic discord and financial insecurity during and prior to the enforced lockdown (with the latter being established retrospectively). Factor 2, *Family and relational stress*, included two items: tensions and conflicts in the family/relationship and stress of cohabitation in a confined space (before vs. during lockdown). Spearman–Brown split‐half coefficient for factor 2 was 0.71 before and 0.88 during the lockdown.

Factors 3 and 4—both single‐item factors—addressed *Financial stress* and a *lack of Time and Space for oneself* (before vs. during lockdown). All items were rated on 4‐point Likert scales (ranging from 0 = not at all to 3 = a great deal), yielding a total of 3 baseline subscores (factors 2–4) and 4 lockdown subscores (factors 1–4) in the range 0–3. A factor analysis confirmed the above‐mentioned structure, whereby the four factors explained a total of 80.06% of the variance for the entire set of variables (with factors 1–4 explaining 28.05%, 31.19%, 11.92% and 8.90% of the variance, respectively). Where dimensions accommodated multiple items (dimensions 1 and 2, respectively), scores were averaged. A total baseline and lockdown stress score was calculated as the sum of factors 2, 3 and 4.

#### 2.3.2 Clinical measure of disease activity

Clinical disease activity was assessed using several questionnaires measuring the activity of IBD during the previous week along with the presence of functional GI symptoms over the preceding 10 days.

##### Patient‐based Simple Clinical Colitis Activity Index

The Patient‐based Simple Clinical Colitis Activity Index (P‐SCCAI, Bennebroek, Nieuwkerk, et al., 2013) is a six‐domain self‐report measure of UC activity. The items evaluate UC symptoms in terms of bowel frequency (day/night), blood in faeces, general well‐being, urgency of defecation and extracolonic features. The first four domains—bowel frequency during the day (scored 0–3), night (scored 0–2), blood in stool (scored 0–3) and general well‐being (scored 0–3)—are all single‐item domains. In contrast, urgency of defecation is composed of three items (scored yes/no = 1/0) while the domain extracolonic features include a total of six items (scored yes/no = 1/0) measuring the manifestation of four extracolonic features (erythema nodosum, arthritis, uveitis and pyoderma gangrenosum). The instrument yields a total CU disease activity score in the range 0–20, with value 2 being the cut‐off point defining active disease (score ≤ 2 = remission; >2 mild to moderate; >6 severe disease activity).

##### Patient Harvey–Bradshaw Index

The Patient Harvey–Bradshaw Index (P‐HBI, Bennebroek, Hoeks, et al., 2013) generates a self‐reported CD activity score and consists of 11 items relating to five domains: general well‐being (1 item, scored 0–4), abdominal pain (1 item, scored 0–3), number of daily liquid stools (open question) and extraintestinal manifestations (8 items relating to arthralgia, uveitis, erythema nodosum, aphthous ulcer, pyoderma gangrenosum, anal fissure, new fistula and abscess, scored yes/no = 1/0). A total P‐HBI score ≥ 5 denotes active disease (whereby 5–7 = mild; 8–16 = moderate and >16 = severe disease).

##### Irritable Bowel Syndrome Symptom Severity Score

The Irritable Bowel Syndrome Symptom Severity Score (IBS‐SSS, Francis et al., 1997) is a five‐item self‐report instrument quantifying functional GI symptoms in terms of severity and duration of abdominal pain, abdominal distension (bloating, swollen, tight tummy), bowel habits, and QoL. Items are rated on visual analogous scales ranging from 0 to 100, generating a maximum IBS‐SSS score of 500 (whereby 75–175 denotes mild; 175–300 moderate and >300 severe cases). Cronbach's alpha for the IBS‐SSS before the lockdown was 0.84 and during the lockdown 0.85.

##### Disease‐related quality of life

Subjective health status was assessed using the Inflammatory Bowel Disease Questionnaire (IBDQ) (Guyatt et al., 1989)—a 32‐item self‐report instrument comprising four subscales, namely GI symptoms (10 items); systemic symptoms (5 items); emotional function (12 items) and social function (5 items) in the past 2 weeks. Items are scored on 7‐point scales (with 1 representing the lowest function and 7 the best function), yielding a total IBDQ score in the range 32–224 (10–70; 5–35; 12–84; 5–35 for the respective subscales) with higher scores denoting a better QoL. Cronbach's alpha for the IBDQ before and during the lockdown was 0.96.

##### Perceived social isolation

To evaluate current and pre‐lockdown (retrospective) social isolation, the University of California Los Angeles (UCLA) loneliness scale was used (Russell et al., 1978), a 20‐item self‐report instrument scored on 4‐point Likert scales (ranging from I never feel this way to I often feel this way). Total loneliness scores (sum of all item scores) range from 20 to 80 with higher scores marking greater loneliness. Cronbach's alpha for the UCLA before the lockdown was 0.97 and during the lockdown 0.94.

### 2.4 Data analysis

Data were first examined for accuracy of data entry and missing or extreme values. One patient, for whom a total P‐HBI score was not available, was omitted from the respective analyses. Main research questions were analysed by means of dependent *t*‐tests and partial correlation analyses, using SPSS 26. First, paired‐sample *t*‐tests were performed to explore possible lockdown‐related changes in IBD disease activity, QoL, total stress experiences and perceived social isolation. Next, separate GLM repeated‐measures ANOVAs were conducted to examine the effect of IBD type (CD, UC) and gender on these lockdown‐related changes. Here, time (before and during lockdown) functioned as within‐subject factor and condition (CD and UC) or gender as between‐subject factor. Since none of these variables significantly influenced interactions regarding lockdown‐related changes in stress experiences, clinical disease activity and disease‐related QoL, they were excluded from the final analyses.

Subsequently, separate partial correlations were conducted to evaluate whether IBD disease activity and QoL during the lockdown were significantly related to lockdown‐related stress experiences (*total stress experiences* or *social isolation*) after correction for stress experiences (total stress or isolation) and disease activity or QoL at baseline. Lastly, partial correlations were computed to evaluate whether COVID‐19 anxiety was significantly related to IBD activity and QoL during the lockdown, whilst correcting for baseline IBD activity or QoL. In case of multiple comparisons, Bonferroni corrections were made. Throughout all analyses, the standard rejection criterion was set at *p* < 0.05 (two‐tailed).

## 3 RESULTS

### 3.1 Disease activity characteristics

Observed disease activity at timepoint 1 (T1; baseline) and 2 (T2; lockdown) is presented in Table 1. At study entry (T1), IBD was active in almost half all CD and approximately two‐thirds of all UC patients (as evident from a P‐HBI score > 5 or a P‐SCCAI score > 2). During the lockdown (T2), this percentage remained fairly stable for CD patients but dropped slightly for UC patients. Additionally, at T1, roughly two‐thirds of all patients presented with functional GI complaints (as evident from an IBS‐SSS score > 74), with symptoms being mild in 3 CD | 8 UC patients, moderate in 13 CD | 9 UC patients and severe in 5 CD | 5 UC patients. At T2, functional GI symptoms were mild in 5 CD | 10 UC patients, moderate in 8 CD | 8 UC patients and severe in 5 CD | 5 UC patients.

**TABLE 1:** Disease activity before and during the lockdown

Variable	CD patients (*n = *30)		UC patients (*n = *33)	
	Baseline	Lockdown	Baseline	Lockdown
Active disease^a^	44.8 (13; 5–21)	46.7 (14; 5–14)	63.6 (21; 3–14)	54.5 (18; 3–12)
Functional GI symptoms^b^	70 (21; 75–360)	60 (18; 85–372)	66.7 (22; 80–396)	69.7 (23; 89–354)
QoL^c^	166.5 (112–214)	168.0 (97–216)	173.0 (69–217)	172.0 (100–216)

Abbreviations: CD, Crohn's disease; GI, gastrointestinal; IBD, inflammatory bowel disease; IBDQ, Inflammatory Bowel Disease Questionnaire; IBS‐SSS, Irritable Bowel Syndrome Symptom Severity Score; P‐HBI, Patient Harvey–Bradshaw Index; P‐SCCAI, Patient‐based Simple Clinical Colitis Activity Index; QoL, quality of life; UC, ulcerative colitis.

^a^ Active disease as evident from a P‐HBI score >5 for CD patients and a P‐SCCAI score >2 for UC patients. One CD patient failed to report a baseline P‐HBI total score: % (*n*; whereof Min–Max).

^b^ Patients experiencing functional gastrointestinal symptoms as evidenced by an IBS‐SSS score >74: % (*n*; whereof Min–Max).

^c^ Disease‐related quality of life as measured with the IBDQ whereby higher scores indicate a better QoL: *Median* (Min–Max).

### 3.2 Main impact of the lockdown

Perceived stress experiences, degree of social isolation, IBD activity and disease‐related QoL at baseline as well as during the lockdown are presented in Table 2. As corona fears were assessed only once (during the lockdown), they were not part of the analyses (*M*: 1.08, SD: 0.72). Paired‐sample *t*‐tests only revealed a significant increase in stress experiences and social isolation during (timepoint 2) compared to before (timepoint 1) the lockdown, suggesting that the lockdown enforced by the Dutch government was associated with a rise in average stress experiences and social isolation without having an overall effect on IBD complaints.

**TABLE 2:** Stress experiences, social isolation, IBD activity, and disease‐related QoL before and during the COVID‐19‐related lockdown^a^

Variable (*n = 6*3)	Baseline		Lockdown		*t* (62)	*p*
	*M*	*SD*	*M*	*SD*		
Total stress experiences	1.44	1.17	2.21	1.82	**−4.804**	**<0.001**
Social isolation (UCLA)	29.30	12.61	33.49	11.77	**−4.766**	**<0.001**
P‐HBI	4.03	3.52	3.92	3.21	0.300	0.765
P‐SCCAI	3.83	3.27	3.86	3.32	**−**0.112	0.911
IBS‐SSS	164.73	118.58	155.24	121.86	0.997	0.322
IBDQ	165.73	34.06	168.17	33.76	**−**0.977	0.323

*Notes:* Total stress experiences = sum of reported family stress, financial stress and lack of time or space for oneself; higher scores indicate more stress experiences. UCLA = University of California Los Angeles loneliness scale; higher scores indicate greater loneliness. P‐HBI = CD activity; higher scores indicate greater disease activity. P‐SCCAI = UC activity; higher scores indicate greater disease activity. IBS‐SSS = functional gastrointestinal symptoms; higher scores denote more symptoms. IBDQ = disease‐related quality of life; higher scores indicate a better quality of life.

Abbreviations: IBD, inflammatory bowel disease; IBDQ, Inflammatory Bowel Disease Questionnaire; IBS‐SSS, Irritable Bowel Syndrome Symptom Severity Score; P‐HBI, Patient Harvey–Bradshaw Index; P‐SCCAI, Patient‐based Simple Clinical Colitis Activity Index; QoL, quality of life.

^a^ Excluding participants with psychiatric comorbidity from analyses yielded similar results.

### 3.3 Impact lockdown‐related changes on IBD activity

As depicted in Table 3, partial correlation analyses were run to evaluate whether the lockdown‐related experiences of stress, social isolation and corona fears were significantly related to lockdown‐related alterations in IBD expression and disease‐related QoL.

**TABLE 3:** Partial correlations between IBD activity and various stress experiences (total stress, social isolation or corona‐fears) during the lockdown controlled for disease activity and the respective stress experience at baseline^d^

Variable	Total stress experiences during the lockdown^a^		Social isolation during the lockdown^b^		Corona fears^c^	
(*n = 6*3)	Partial r	*p*	Partial *r*	*p*	Partial *r*	*p*
P‐HBI	0.315	0.303	**0.319**	**0.013**	**0.307**	**0.016**
P‐SCCAI	**−**0.039	0.765	0.056	0.668	0.181	0.158
IBS‐SSS	**0.342**	**0.007**	**0.254**	**0.048**	0.201	0.117
IBDQ total score	**−**0.189	0.145	**−0.465**	**<0.001**	**−**0.229	0.073
IBDQ GI function	**−**0.145	0.279	**−0.318**	**0.013**	**−**0.147	0.255
IBDQ systemic function	**−**0.204	0.114	**−0.432**	**0.001**	**−**0.228	0.075
IBDQ emotional function	**−**0.167	0.199	**−0.448**	**<0.001**	**−**0.221	0.084
IBDQ social function	**−**0.129	0.322	**−0.501**	**<0.001**	**−0.322**	**0.011**

*Notes:* P‐HBI = assessment of CD activity. P‐SCCAI = assessment of UC activity. IBS‐SSS = index of functional gastrointestinal (GI) symptoms. IBDQ = disease‐related QoL.

Abbreviations: GI, gastrointestinal; IBD, inflammatory bowel disease; IBDQ, Inflammatory Bowel Disease Questionnaire; IBS‐SSS, Irritable Bowel Syndrome Symptom Severity Score; P‐HBI, Patient Harvey–Bradshaw Index; P‐SCCAI, Patient‐based Simple Clinical Colitis Activity Index; QoL, quality of life.

^a^ Partial correlation controlled for stress experiences and symptom severity at baseline.

^b^ Partial correlation controlled for degree of social isolation and symptom severity at baseline.

^c^ Partial correlation controlled for symptom severity at baseline.

^d^ Excluding participants with psychiatric comorbidity from analyses yielded similar results except for the correlation between IBS‐SSS and social isolation (partial *r *= 0.233, *p* = 0.079)—most likely due to limited statistical power.

#### 3.3.1 Functional gastrointestinal complaints

In line with our expectations, functional GI symptoms (IBS‐SSS scores) during the lockdown were positively associated with experiences of social isolation (UCLA) and stress during the lockdown (after correction for baseline *symptoms* and *social isolation* or *stress*), suggesting that a lockdown‐related increase in social isolation as well as an increase in experiences of stress coincided with an exacerbation of functional GI symptoms in IBD patients (Table 2). To determine which stressor (i.e., family stress, a lack of time, and space for oneself or financial stress) of the composite overall stress score was most fundamental to the association between stress and functional GI symptoms, partial correlations between IBS‐SSS scores and the respective stressors were computed (corrected for baseline stress and IBS‐SSS scores). From this it emerged that symptom severity during the lockdown was significantly related to family stress (partial *r = *0.319, *p = *0.012) and lack of time and space (partial *r = *0.261, *p = *0.042), but not to financial stress during the lockdown (partial *r = *0.223, *p = *0.084), reflecting that lockdown‐related changes in functional GI symptoms were primarily provoked by changes in interrelational dynamics rather than changes in financial hardship. No significant partial correlations between function GI symptoms and corona fears were observed (partial *r* = 0.201, *p* = 0.117).

#### 3.3.2 Clinical disease activity (CD and UC)

Both lockdown‐related social isolation (corrected for baseline isolation and symptoms) and presence of corona fears (corrected for baseline symptoms) were positively related to CD activity during the lockdown, demonstrating that the pandemic‐evoked increase in feelings of loneliness and corona fears coincided with an increase in CD activity. No significant relationships between the different stressors and UC index scores were detected.

#### 3.3.3 Disease‐related QoL

From the partial correlation analyses it emerged that lockdown‐related experiences of social isolation (UCLA) were inversely related to all dimensions of Qol during the lockdown (corrected for baseline isolation and QoL), as was the manifestation of corona fears to social impairment during the lockdown (corrected for baseline social function).

## 4 DISCUSSION

The current prospective study investigated whether the COVID‐19 pandemic was associated with a change in emotional stress experiences in corona‐free IBD patients, and as a function thereof with a shift in GI symptomatology and QoL. The results are partially confirmatory. First, the pandemic did indeed mark a significant increase in overall stress experiences in IBD patients which—in turn—corresponded to an exacerbation of primarily functional GI symptoms. Moreover, the enforced lockdown came with a sharp increase in experiences of social isolation and loneliness—which correlated not only with an aggravation of functional GI symptoms but also of CD activity and overall dysfunction. Lastly, corona anxiety was significantly related to CD activity and social dysfunction. As such, the data emphasize the additional impact of the COVID‐19 pandemic on GI complaints in IBD patients, most likely via the promotion of negative affective experiences.

### 4.1 Effect of lockdown‐related stress experiences on IBD activity

It is increasingly recognized that emotional stress is a major trigger of symptoms in IBD patients (Camara et al., 2009; Hisamatsu et al., 2007; Maunder & Levenstein, 2008; Mawdsley & Rampton, 2005). Although this relationship remains to be more thoroughly examined, the present study lends some support to this idea by establishing that lockdown‐related changes in emotional distress coincided with a change in GI symptoms. That is, when the lockdown was accompanied by an increase in stress experiences (in 50.8% of all participants respectively), a worsening of mainly functional GI complaints was observed, while a decrease in stress experiences (observed in 11.1% of all participants) coincided with a drop in functional GI symptoms. Interestingly, of all stressors analysed, the worsening of functional GI symptomatology was primarily related to an escalation of interpersonal challenges (tensions and conflicts in the family or romantic relationships) along with a growing loss of life‐space (lack of time/space for oneself). They, therefore, appear to confirm previous findings showing that excessive interpersonal closeness is detrimental to a person's self‐regulating capacity and associated with an increased risk of interpersonal conflicts (Jones, 1975) which in turn may exacerbate functional GI symptoms (Gerson et al., 2006; Herzer et al., 2011). Furthermore, the present data are also consistent with previously established pro‐inflammatory effects of interpersonal stress from romantic partners, family members and friends (Allen et al., 2018; Kiecolt‐Glaser et al., 2005; Miller et al., 2009; Yang et al., 2014).

Relational theory seems particularly apt to elucidate these findings. First, invasion of personal space and conflicts amongst romantic partners are known to jeopardize a person's sense of security and activate neuroendocrine‐immune pathways, thereby upregulating intestinal inflammation, altering gut function (Hänsel et al., 2010; Kiecolt‐Glaser & Newton, 2001; Kiecolt‐Glaser et al., 1996, 2005; Norman et al., 2012; Robles & Kane, 2014) and increasing gut permeability (Kiecolt‐Glaser et al., 2018). Second, relational conflicts and other forms of contact disturbance may undermine the homeostatic regulatory functions of the relationship (interpersonal regulation of psychophysiological arousal, dyadic coping) thus compromising one's ability to cope with distressing experiences (Butler & Randall, 2013; Field, 2011; Sbarra & Hazan, 2008; Volet et al., 2009). Lastly, literature shows that the interpersonal styles predominantly observed in IBD patients (anxious or avoidant attachment styles) (Agostini et al., 2010, 2014, 2016) interfere with adaptive interpersonal regulation and are associated with abnormal affective, neuroendocrine and inflammatory responses to interpersonal conflicts (Diamond et al., 2006; Ehrlich, 2019; Gouin et al., 2009; Maunder & Hunter, 2001; Pietromonaco, DeBuse, et al., 2013; Pistole, 1994) increased immune changes during periods of altered social function (Gouin & MacNeil, 2019), enhanced basal systemic inflammation (Pietromonaco, Uchino, et al., 2013) as well as heightened symptom neglect and medication non‐adherence (Agostini et al., 2019; Colonnello & Agostini, 2020) all of which have a detrimental effect on IBD management.

### 4.2 Effect of stress from lockdown‐related social isolation and loneliness on IBD activity

Next, we explored the effect of a lockdown resultant increase in stress from social isolation on IBD activity. The outcome was particularly remarkable, revealing that stress from loss of interpersonal connectedness was also directly related to IBD activity. That is, greater social isolation during the lockdown coincided with stronger CD activity, more functional GI symptoms and lower subjective health. As to the latter, participants experiencing higher levels of loneliness and social isolation reported greater social and emotional dysfunction, more systemic symptoms and more complaints directly related to their bowel disruption. These observations are in line with previous findings showing that stressful social experiences (especially threats to social connectedness) deeply interfere with health‐relevant physiological processes – in particular inflammatory dynamics (Cacioppo & Hawkley, 2003; Cole et al., 2007; Eisenberger & Cole, 2012; Eisenberger et al., 2017; Moieni et al., 2015; Seeman, 1996) – and increase the risk of chronic inflammation‐related diseases (Cohen et al., 1997; Caspi et al., 2006; Kroenke et al., 2006). Similarly, they are consistent with the notion that organisms easily get dysregulated and symptomatic when denied interaction and coregulation – an effect that may even extend to those with properly developed self‐regulatory abilities and points to the significance of social interaction dynamics for biological function (Hofer, 1984; 1994).

### 4.3 Effect of stress from COVID‐19 fears on IBD activity

Lastly, we explored whether COVID‐19‐related anxiety contributed to a further exacerbation of IBD symptoms and/or a decrease in QoL. From the results it emerged that the manifestation of corona fears was, indeed, accompanied by stronger CD activity and greater social impairment. This is in accordance with previous studies identifying anxiety as the factor primarily involved in the association between stress and the exacerbation of CD activity (Cámara et al., 2011) and those linking depression and anxiety to clinical recurrence of IBD (Mikocka‐Walus et al., 2016). Given the accumulating evidence for a strong interconnectivity between immune and affective states (Maydych, 2019), it seems plausible that above‐referenced relationship between anxiety and IBD activity is reciprocal in nature, with anxiety not only promoting GI symptoms, but intestinal inflammation itself reinforcing negative affectivity and stress sensitivity (van den Brink et al., 2018). In support thereof, it has been shown that heightened inflammatory activity coincides with greater attention to negative information and stronger affective and physiological reactivity to negative information (Bruch, 2016; Dooley et al., 2018; Goehler et al., 2007; Maydych, 2019; Reichenberg et al., 2001).

In conclusion, the present data indicate that stress resulting from COVID‐19 anxiety was significantly associated with CD activity and overall dysfunction, whereby it is feasible that the bowel symptoms themselves accentuated subjects' stress and anxiety sensitivity.

### 4.4 Limitations and conclusion

When evaluating the results of this study, several shortcomings call for reflection. First, the data were derived from a relatively small number of patients and necessitate replication in larger samples. Despite that, a significant effect of stress (loss of personal space, relational conflicts, social isolation and corona fears) on GI symptomatology was found. Still, the limited number of participants might explain why changes in stress experiences were not significantly related to variations in UC activity, while such a connection has been established by others. Moreover, a larger sample size is needed to further investigate any group differences in the associations found. Second, the degree of stress and isolation prior to the lockdown were evaluated retrospectively and are therefore potentially distorted due to recall bias, though the direction of the effect was in line with earlier observations. Third, disease monitoring was restricted to subjective self‐reports as the COVID‐19‐related measures precluded comprehensive patient monitoring in research labs and university clinics. In follow‐up research, subjective measures of disease activity might be enriched with objective assessments (i.e., endoscopic assessment, biomarkers of inflammatory activity) whereby the number of measuring points could be expanded as well to better determine when changes in disease activity occur—relative to changes in interpersonal relatedness—and when they diminish. This is all the more relevant in light of the possibility that self‐reports of disease activity are themselves affected by stress and perceived social isolation, while—conversely—feelings of stress and social isolation are fuelled by perceived disease burden. Lastly, alternative explanations for the observed shifts in symptom patterns, such as lockdown‐related shifts in nutritional intake and self‐care, cannot be ruled out.

For now, the present study established that shifts in IBD expression are closely related to changes in emotional stress experiences and social interaction dynamics whereby particularly a drift towards ‘too much interpersonal proximity’ (loss of time and space for oneself) or ‘too much interpersonal distance’ (perceived social isolation, loneliness) coincides with a flare up of symptoms and increase in overall dysfunction. As such, it confirms that the human self, for its stability and regulation, must balance its need for closeness (feeling related to, connected with and open towards the world and others) with its need for distance (being a subject of its own, distinct and independent from others) (Galbusera et al., 2019). Furthermore, the results show that psychological (affective) and intestinal states are strongly interrelated, pointing to the integrative and reciprocal rather than separative nature of psyche‐soma. It follows that, in order to better control temporal fluctuations in IBD expression, interventions aimed at alleviating psychological stress (particularly anxiety symptoms and relational tensions) and enhancing interpersonal distance regulation (allowing the patient to better negotiate the tension between processes of distinction and connection in intimate relationships) may offer an effective therapeutic approach worth further exploration.

## CONFLICT OF INTEREST

The authors do not report any conflict of interest.

## AUTHOR CONTRIBUTIONS

All authors participated in designing the study and analysing the data. Boukje Yentl Sundari Nass collected the data and drafted the manuscript, which was then critically revised by Rob Markus and Pauline Dibbets. The final document was approved by all authors.

## Data Availability

The data underlying this article will be shared on reasonable request to the corresponding author.
